# Three-Dimensional Blood-Brain Barrier Model for *in vitro* Studies of Neurovascular Pathology

**DOI:** 10.1038/srep15222

**Published:** 2015-10-27

**Authors:** Hansang Cho, Ji Hae Seo, Keith H. K. Wong, Yasukazu Terasaki, Joseph Park, Kiwan Bong, Ken Arai, Eng H. Lo, Daniel Irimia

**Affiliations:** 1 BioMEMS Resource Center, Massachusetts General Hospital, Harvard Medical School, Boston, Massachusetts 02129, United States; 2 Neuroprotection Research Laboratory Center, Mass General Hospital, Harvard Medical School, Charlestown, 02129, United States; 3 Mechanical Engineering and Engineering Science, Center for Biomedical Engineering and Science, University of North Carolina at Charlotte, 28223, United States

## Abstract

Blood–brain barrier (BBB) pathology leads to neurovascular disorders and is an important target for therapies. However, the study of BBB pathology is difficult in the absence of models that are simple and relevant. *In vivo* animal models are highly relevant, however they are hampered by complex, multi-cellular interactions that are difficult to decouple. *In vitro* models of BBB are simpler, however they have limited functionality and relevance to disease processes. To address these limitations, we developed a 3-dimensional (3D) model of BBB on a microfluidic platform. We verified the tightness of the BBB by showing its ability to reduce the leakage of dyes and to block the transmigration of immune cells towards chemoattractants. Moreover, we verified the localization at endothelial cell boundaries of ZO-1 and VE-Cadherin, two components of tight and adherens junctions. To validate the functionality of the BBB model, we probed its disruption by neuro-inflammation mediators and ischemic conditions and measured the protective function of antioxidant and ROCK-inhibitor treatments. Overall, our 3D BBB model provides a robust platform, adequate for detailed functional studies of BBB and for the screening of BBB-targeting drugs in neurological diseases.

Endothelial cells in the brain form an impermeable barrier, which delineates a unique chemical, functional, and immunologic environment in a central nervous system (CNS)^1^,^2^. The tight junctions between endothelial cells in the blood-brain barrier (BBB) allow only small nutrient molecules and gases to diffuse across the BBB and limit the entrance of larger, potentially neurotoxic macromolecules, bacteria, and leukocytes from the blood^3^,^4^. Recent studies reported that various neuropathology including neuroinflammation^5^ and cerebral ischemia^6^,^7^,^8^ can induce the loss of the tightness or the destruction of BBB, allowing the leakage of serum proteins and the entrance of blood cells into the brain tissue. These processes could have relevance to various neurological diseases including Alzheimer’s disease (AD)^9^,^10^, Parkinson’s disease (PD)^11^,^12^,^13^, amyotrophic lateral sclerosis (ALS), brain edema, dementia, and multiple sclerosis^14^,^15^,^16^. Despite their importance for health and disease, studies that disrupt BBB in animal models pose significant challenges. The complexity of brain microenvironment reduces the ability to isolate the specific roles of the endothelial cells modulating the BBB tightness during neurovascular disorders from various factors in the blood stream or CNS. *In vitro* BBB models provide microenvironment conditions that are usually easier to control and quantify. However, their relevance is often limited due to over-simplification. They rely on 2 dimensional cellular contructs and are valuable only for end-point assays and the study of simple stimuli^17^,^18^. Recent models, relying on microscale technologies, could manipulate vertically stacked and planar BBB models, with limited capability for visualizing cellular interaction across the BBB^5^,^19^,^20^.

Here, we construct an *in vitro* BBB model by arranging endothelial monolayers in tube-like structures on a single-layered microfluidic platform^21^. We validate the tightness of the constructed model by visualizing the delayed dye leakage across our BBB, the expression of endothelial junction proteins, Zonula Occluden-1 (ZO-1) and VE-Cadherin along the cellular boundaries on top/bottom membranes, and by observing the blockage of neutrophil migration across the side membrane in the presence of standard chemoattractant. We demonstrate the potential relevance of this model in studying neuroinflammation and cerebral ischemia, by measuring the effect of antioxidant scavenger, edaravone^22^,^23^,^24^, and Rho Kinase (ROCK) inhibitor, Y-27632^25^,^26^, on BBB permeability and protection against hypoxia-inducing oxidative damage.

## Results and Discussion

**Figure 1:**
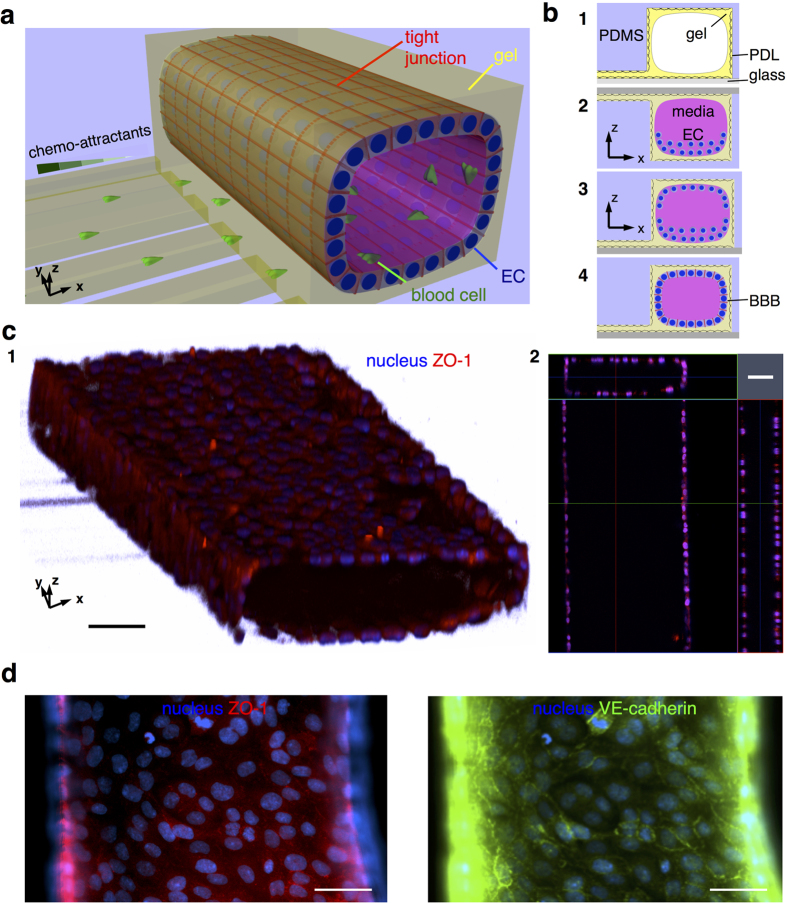
Schematic representation of a 3D *in vitro* BBB model and structural details. (**a**) A 3D BBB model consists of endothelial cells (EC) arranged in a cylindrical monolayer, forming a tight seal, and separating a “blood” compartment from an array of capillaries presenting chemotactic gradients, which simulate white blood-cell transmigration across the BBB. (**b**) The 3D BBB model is constructed by (**1**) coating an adhesive molecules, Poly-D-lysine (PDL), and a gel on polydimethyl-siloxane (PDMS) microstructures and a glass substrate, (**2**) plating brain endothelial cells (EC) first on the top and (**3**) later on the bottom surfaces, and (**4**) culturing to form tight monolayers. (**c**) The constructed BBB model has a lumen lined by confluent monolayers of endothelial cells on all surfaces, which resembles *in vivo* BBB. Prospective **(1)** and cross-sectional **(2)** views are presented. (**d)** The formation of a tight BBB is confirmed by the imaging of localized tight junction-involving proteins along cellular boundaries. Fluorescent Zonula Occluden-1 (ZO-1) and Ve-Cadherin proteins are shown in left and right panels, respectively. Scale bars, 50 *μ*m.

**Figure 2:**
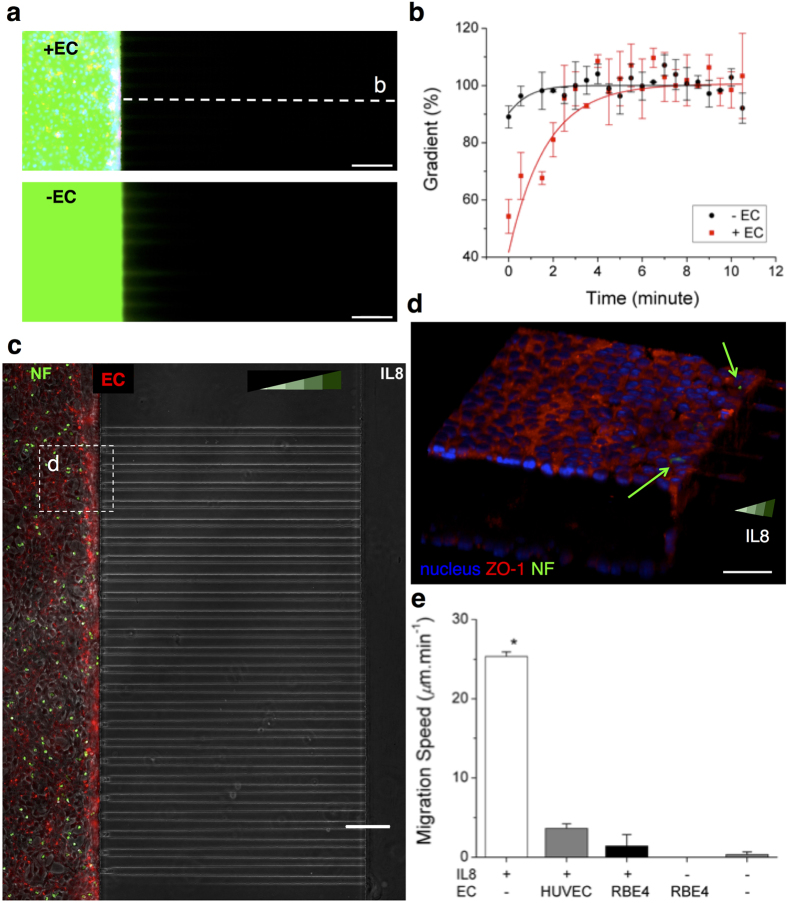
Constructed 3D BBB model shows reduced dye leakage and inhibited penetration of white blood cells. (**a**) The diffusion of FITC-dextran (M.W. 40 kDa) is significantly prevented for the first several minutes through the rat brain endothelial cells (EC) that form the BBB model (top). In the absence of the EC barrier, the fluorescent dextran diffuses into the side channels (bottom). (**b**) The EC layers significantly reduce the dye leakage and slow the formation of a gradient along channels next to the EC-loaded, left compartment. (**c**) The BBB model was evaluated for its ability to block the penetration of human neutrophils in the presence of BBB-permeable chemoattractant Interleukin 8 (IL-8) in the right compartment. Neutrophils (NFs) were stained in green and loaded inside the left compartment. No neutrophils were observed to cross the BBB model. (**d**) The neutrophils enclosed with the EC layers are imaged in a confocal microscope. (**e**) Neutrophil migration along the IL-8 gradient is significantly inhibited and consequently averaged migration speed becomes nearly zero in the presence of the EC layers. (one-way ANOVA. *P < 1 × 10^−7^ by Tukey’s post-hoc test in comparison with other conditions). n_cell_ = 18 for each condition. Data represent mean ± s.e.m. Scale bars, 50 *μ*m.

**Figure 3:**
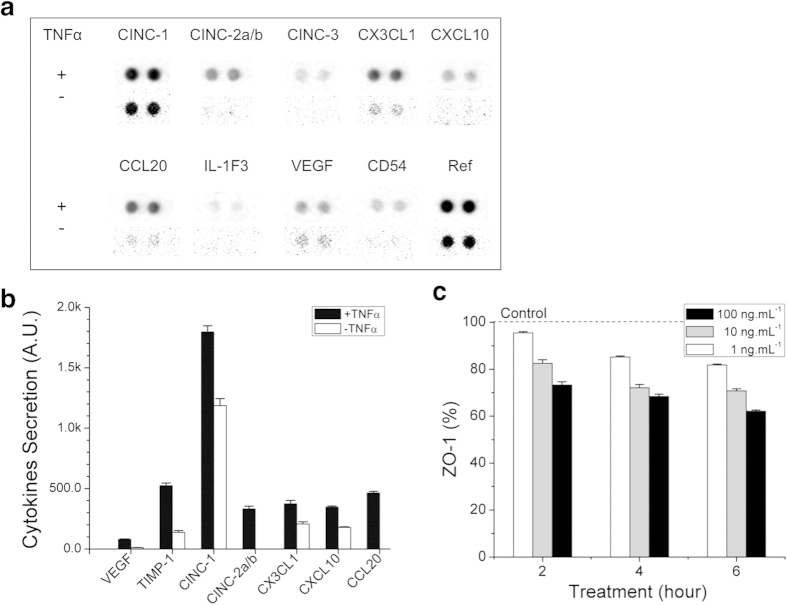
BBB model response to neuroinflammation stimulus. (**a**) The changes in the levels of twenty-nine cytokines were measured after stimulations of the BBB model with tumor necrosis factor alpha (TNF-α, upper row) compared to unstimulated baseline (second row). (**b**) Seven inflammation involving cytokines were noticeably further secreted by the BBB model with TNF-α treatment [100 ng.mL^−1^] for 24 hours. (**c**) The levels of ZO-1 decreased with increasing treatment duration and concentration of TNF-α. The levels of ZO-1 decrease to 60% of control after 6 hours of exposure to TNF-α [100 ng.mL^−1^]. The dotted line represents the value of ‘Control’ group (no treatment). n_cytokine_ = 4, n_cell_ = 20 for each condition. Data represented as mean ± s.e.m.

**Figure 4:**
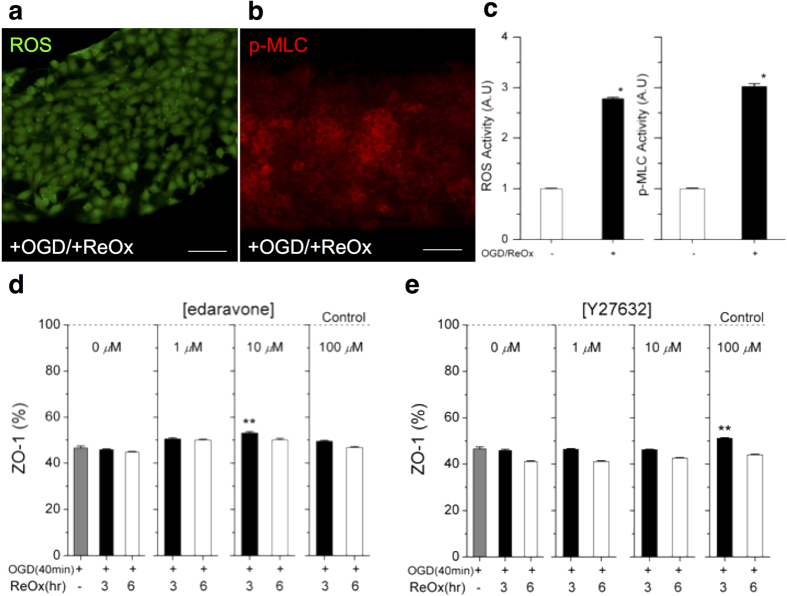
BBB model recovery after ischemia and the effect of protecting drugs. Ischemia is simulated by a two-step procedure of oxidative stress: oxygen-glucose deprivation (OGD) followed by reoxygenation (ReOx, abrupt supply of oxygen causing oxidative stress). The oxidative damage to the BBB model is confirmed by changes in the levels of (**a**) Reactive Oxygen Species (ROS) and (**b**) Rho Kinase (ROCK) activities. ROCK activity is evaluated from the change in phosphorylation of myosin light chain (p-MLC), which is upregulated by activated ROCK. (**c**) The expression of ROS and p-MLC increases three fold after oxidative stress compared to the absence of the stress. (**d**) The reduction of ZO-1 was partially prevented with the treatment of an antioxidant, edaravone [10 *μ*M], from 46% to 53%. (**e**) The ROCK inhibitor protects ZO1 by 10 % with the treatment of Y27632 [100 *μ*M] at 3 hours of reoxygenation. Student T-Test, two-tailed hypothesis, *P < 0.05 with no ischemia treatment, **P < 0.05 with no drug treatment, n_cell_ = 64 for each condition. Data represent mean ± s.e.m. Scale bars, 50 *μ*m.

We constructed our 3D BBB model in the form of a tube inside a single layered microfluidic platform. The tube of endothelial cells inside the platform is geometrically similar to small brain blood vessels. The single-layered platform is convenient for integrating with other functional components and assays, *i.e.* chemotactic transmigration (Fig. 1). Our current model is static, with no fluid flow and no shear stress on the constructed model during culturing. To achieve faster maturation of barrier function, cells were seeded at high density on both the top and bottom surfaces and were confined along the compartments through the use of surface tension at the ends (Figs 1b, 2, 3, Supplementary Fig. S1). The seeded cells formed strong attachment to the thin gel layer on microstructured PDMS substrate (Supplementary Fig. S2) and formed tight monolayers two or three days after plating. We validated the formation of endothelial monolayers on 3D surfaces and the tightness of the junctions between adjacent cells by imaging each surface (Figs 1b, 4, supplementary Fig. S3). The confocal images showed a 3D membrane (Fig. 1c, 1) and monolayers of endothelial cells on all surfaces (Figs 1c, 2). The tightness of the BBB was confirmed by immunostaining of proteins: ZO-1 and VE-Cadherin, which are known to be responsible for the integrity of the BBB^27^,^28^. The tightness of the BBB was confirmed by immunostaining of proteins representative for the BBB tightness: ZO-1 and VE-Cadherin. We found ZO-1 and VE-cadherin expressed selectively along the cellular boundaries, in representative patterns (Fig. 1d). The localization of expressed ZO-1 and VE-Cadherin on our model was not as clear as other endothelial monolayers on thin gel-coated or flat substrates, probably due to relatively thicker and softer gel compared to that reported in other literature^29^. Despite this limitation, our 3D BBB model could still be a convenient platform for various assays utilizing various surfaces: top and bottom surfaces for evaluating pathology-related assays destructing the BBB tightness, side surfaces for monitoring real-time penetration across the BBB upon the tightness destruction, and a single-layered platform for providing simple integration with other components.

The constructed BBB model could separate an outer (neural) environment from an inner (blood) environment. To demonstrate the separation, we first probed the transport of a dye across the endothelial layer through the side surfaces (Fig. 2). FITC-conjugated dextran at 40 KDa (D1844, Molecular Probes, Inc., Eugene, OR) was introduced into the lumen of the BBB model (Fig. 2a). While the dye penetrated across the endothelial layer, we evaluated the gradient of the dye priming into gel-filled channels outside. The outward flux of the dye was slower in the presence of the endothelium. The gradient peaked at about 7 minutes in the presence of the BBB, compared to less than 4 minutes when no barrier was present (Fig. 2b).

We also probed the transmigration of neutrophils across the endothelial layer, through the side surfaces, in response to a typical chemoattractant, IL8. Neutrophils plated inside the “blood” compartment, become activated, rapidly changed their shape, and were observed moving under the effect of the chemoattractant. However, after two hours, we observed no neutrophils passing through the BBB and reaching the “neural” compartment and the migration channels (Fig. 2c and Supplementary information Movie M1, M3). In the absence of endothelial layers, neutrophils showed massive migration response, along the gradients of IL-8. In control experiments, few neutrophils could cross the layer of HUVEC. (Supplementary information Movie M2, M4, Fig. S4).

The inhibition of neutrophil transmigration by the BBB was also confirmed by confocal microscope imaging (Fig. 2d). In the absence of an endothelial cell layer, the migration speed of fluorescently tagged neutrophils along the gradient of IL-8 of 100 nM reached 25.3 ± 0.6 *μ*m.min^−1^ from the observation for 20 minutes. In the presence of endothelial layer, only one out of 300 neutrophils entered the “neural” compartment after 90 minutes and average speed was significantly lower, at 1.4 ± 1.4 *μ*m.min^−1^. In negative control experiments, in the absence of IL-8, one neutrophil entered the “neural” compartment, migrated spontaneously into one channel (0.3 ± 0.3 *μ*m.min^−1^), and returned back to the “blood” compartment (Fig. 2e).

To demonstrate the utility of the *in vitro* BBB model for probing the effect of neuroinflammation, we treated the constructed BBB with TNF-α (Fig. 3a), which has been previously reported to mediate of neuroinflammation and was detected in patients with neurological disorder at ~1 ng/mL^30^,^31^,^32^. In order to confirm the induction of neuroinflammation by TNF-α treatment in the BBB model, we treated the BBB model with TNF-α at 100 ng.mL^−1^ for 24 hours, extracted conditioned media from the “blood” compartment, and measured the changes in cytokine levels in the media in the “blood” compartment by using a 29-cytokine kit (ARY008, The Proteome Profiler Rat Cytokine Array Panel A Kit, R&D Systems, Inc.). We observed that TNF-α treatment elevated the release of several cytokines, including VEGF^33^, TIMP1^34^, CINC1^35^, CINC2–a/b^36^, CX3CL1^37^, CXCL-10^38^, CCL20^39^, which are known to mediate a neuroinflammatory response compared to control group (no treatment) and confirmed that TNF-α treatment effectively triggered inflammatory effect on our constructed BBB model (Fig. 3a,b). We further incubated the constructed BBB with TNF-α of 0/1/10/100 ng.mL^−1^ for various periods to evaluate its utility as neuro-inflammation model. We observed that ZO-1 proteins, which were highly expressed particularly along the cellular boundary before the TNF treatment, were reduced or even depleted in many regions after the treatment. TNF-α not only reduced the protein levels on the entire regions but also delocalized ZO-1 on the cellular boundary (supplementary Fig. S5). A box plot of averaged peak intensity indicated that BBB destruction was proportional to both of treatment time and the TNF-α concentration (*i.e.* TNF-α at 1 ng.mL^−1^ for 6 hours or TNF-α at 100 ng.mL^−1^ for 3 hours degraded ZO-1 by about 20%) and TNF-α at 100 ng.mL^−1^ for 6 hours could reduce the level of ZO-1 by about 40% (Fig. 3c). To confirm that the disruption of the BBB post TNF-α treatment was not due to cell death, we verified the viability of the endothelial cells by standard assays (supplementary Fig. S6)^40^,^41^. These results imply that neuroinflammation could increase the BBB permeability, favoring the leakage of inflammatory cytokines from/to the brain tissue. Our BBB model could be a convenient platform to systematically evaluate the destructive effects of various neuroinflammatory mediators on the integrity of the BBB model.

To demonstrate that the platform could be used as an ischemia model, we treated the constructed BBB with the OGD procedure (known for the ability to deplete oxygen and glucose) followed by reoxygenation, which induced oxidative stresses and destructed the integrity of the BBB^42^. In order to confirm that the activation of ROS and ROCK by the oxidative stress, we subjected the constructed model to the OGD procedure for one hour, followed by reoxygenation for three hours, and then immunostained the cells for measuring ROS (Fig. 4a, supplementary Fig. S7). We also measured ROCK activity by phosphorylation of myosin light chain (p-MLC), which is upregulated by activated ROCK, within the endothelial cells (Fig. 4b, supplementary Fig. S8). We observed elevated level of ROS and p-MLC, by 2.8 and 3.0 fold, respectively, confirming the effect of oxidative stress on ROS production and ROCK activation in our BBB model (Fig. 4c). After an exposure to OGD procedure for up to an hour and following reoxygenetion for up to 3 hours, the viability decreased by less than 1%, as measured by propidium iodide-staining (supplementary Fig. S9). However, the levels of ZO-1 expression were significantly reduced by more than the half (Fig. 4d,e, supplementary Fig. S10). To restore BBB integrity, a clinically used antioxidant, edaravone and a ROCK inhibitor, Y-27632, which are known to protect BBB against oxidative stresses, were tested on the platform. The addition of edaravone and Y-27632 elevated the level of ZO-1 by 7 to 10% at three-hour after reoxygenation. At 6 hours after reoxygenation, cellular death increased by an additional 1% and ZO-1 markers further reduced by 5%. These effects occurred after the addition of the protective compounds, suggesting limited protective effects of these compounds. The reason for the relatively low protection may be due to the fact that ischemic stress occurs not only due to oxidative stress but also due to hypoxia and other inflammatory effects. We envision that our platform could be employed for screening new compounds, which remove or counteract neurotoxic agents, including reactive oxygen species produced after ischemia or stroke. Our platform may also be useful for studying the contribution of various transporters^43^ and the emerging role of transcytosis systems^44^ to the functionality of BBB in health and disease. The platform may be further improved by the co-culture of astrocytes and pericytes with the BBB^45^ and by exploring the role of flow in the maintenance of BBB tightness over time^46^.

## Conclusions

We designed a microfluidic platform that replicates several mechanical and functional features of the BBB in 3D. We validated the tightness of the BBB model by the presence of membrane proteins localized along cellular boundary, by measurements of dye transport, and by the blocking of neutrophil transmigration across the BBB in the presence of chemoattractants. The tight BBB in our model could be disrupted by exposure to TNF-α and in conditions of ischemia. Overall, this platform could be useful to study the roles of BBB in various diseases and for screening drugs candidates to modulate neuroinflammation and its consequences on BBB^47^.

## Methods

### Fabrication of microstructure and customized wells

Negative photoresists, SU-8 5 and SU-8 50 (MicroChem, Newton, MA), were sequentially patterned using standard lithography on a 4 silicon wafer to create reversed features for cell migration channels and endothelial compartments. The height of these structures is 5 *μ*m and 50 *μ*m in height, respectively. A mixture of a base and a curing agent with a 10:1 weight ratio (SYLGARD 184 A/B, Dowcorning, Midland, MI) was poured onto the SU-8 mold and cured at room temperature under vacuum for one hour and, subsequently, in an oven at 80 °C for more than 3 hours. The cured PDMS was punched for fluid reservoirs and glued by uncured PDMS to a customized acrylic well plate of 6 mm in thickness for the extension of the medium reservoirs, which was machined by a laser-cut (Zing 24, Epilog Laser, Golden, CO) and then incubated at 80 °C overnight for bonding. The composite assembly was bonded to a glass-bottomed multiwell (P06-1.5-20-F, MatTek Corporation, Ashland, MA) or a glass-bottomed UniWell plate (MGB001-1-2-LG, Matrical Bioscience, Spokane, WA) using oxygen plasma at 50 mW, 5 ccm, for 30 seconds (PX-250, March Plasma Systems, Petersburg, FL)^21^.

### Surface treatment of PDL and gel

Immediately after bonding, 10 *μ*L of poly (D-lysine) solution (PDL, M.W. 70,000–150,000, 1.0 mg.mL^−1^, Sigma-Aldrich, St. Louis, MO) was injected into the each compartment and incubated at room temperature to promote cell adhesion. After 2 hours, we rinsed with autoclaved and 0.2 *μ*m filtered water (AM9920, Life Technologies, Grand Island, NY). The PDL-coated platforms were filled with pH 7.4 collagen Type 1 (354249, Becton Dickinson, Franklin Lakes, NJ) at 2 mg. mL^−1^ and incubated at 37 °C for 30 minutes. The cured collagen in the endothelia compartments was replaced by a cell culturing medium (EGM-2 MV BulletKit: CC-3156 & CC-4147, Lonza Walkersville) supplemented with 1% P/S for overnight to promote the cellular growth. The flow of media was such that the gel in smaller migration channels remains inside the channels, to prevent spontaneous entrance of endothelial cells during plating.

### Endothelial cell preparation and experiment

Medium including rat brain endothelial cells (RBE4, a cell line developed by INSERM^1^) at 60 million cells.mL^−1^ was loaded only in the compartments by removing an extra from both reservoirs and confining them within the compartments with a surface tension. The loaded cells adhered to the upper surface of the compartments when the platforms were placed in an inverted position for two hours in a culturing incubator at 37 °C supplied with 5% CO_2_. After the initial attachment to the upper surface, freshly isolated cells were gently reloaded and then attached to the lower surface at a normal position for two hours. After the attachment to both surfaces, an extra fresh medium of 100 *μ*L was supplied to one of reservoirs and the customized wells and incubated for overnight. The supply of fresh medium was maintained by adding new medium of 100 *μ*L from one side and removing old one from the other side every day. For a neutrophil transmigration study, before experiments, cell membrane was labeled with a red fluorescent dye (PKH26PCL, Sigma-Aldrich) and nucleus with Hoechst 33342 (R37605, Life Technologies) following the manufacturer’s protocol and plated on only one compartment. The constructed 3D BBB was visualized with a confocal microscopy (LSM 710, Carl Zeiss, Thornwood, NY).

### Neutrophil preparation and experiment

Human neutrophils were isolated from whole blood using EasySep Human Neutrophil Enrichment Kits (19257, STEMCELL Technologies, Vancouver, Canada) following the manufacturer’s protocol. After isolation, the cell membrane was stained with green fluorescent dye (PKH2 PCL, Sigma-Aldrich) following the manufacturer’s protocol. The stained neutrophils were re-suspended at a concentration of 10 million cells.mL^−1^ in a culturing medium, Iscove’s Modified Dulbecco’s Media (30-2005, American Type Culture Collection, Manassas, VA) supplemented with 20% FBS and 1% P/S. Chemokine for neutrophils, interleukin 8 (IL-8, 208-IL, R&D Systems, Inc., Minneapolis, MN) of 100 nM was added to the other compartment than an endothelial compartment and incubated for 2 hours to prime IL-8 along the gel-filled migration channels and the endothelial compartment. Stained neutrophils (NFs) at 10 million cells.mL^−1^ were injected into the endothelial compartment and incubated at 37 °C supplied with 5% CO_2_ for 10 minutes before imaging. The neutrophils were cultured in a fully automated microscope (Eclipse Ti, Nikon Inc., Melville, NY) integrated with a heated incubating stage (LiveCell 05-11-0032 Rev B, Pathology Devices Inc., Westminster, MD), which was set at 37.7 °C, 5% CO_2_, and 85% humidity, digitized in a real time (NIS Elements, Nikon Inc.), and the migration speed was calculated by counting the first neutrophil entering each migration channel using Image J.

### TNF-α treatment

To mimic a neuroinflammatory condition, TNF-α (tumor necrosis factor-α, 210-TA, R&D Systems, Inc.) was treated on a constructed BBB model. Brain endothelial cells were plated on a gel-coated microstructure and cultured for two or three days to fully develop monolayers in a normal culturing medium. Cells were further incubated in a culturing medium including TNF-α at various concentrations up to 6 hours for disrupting ZO-1 expression and 24 hours for measuring induced cytokines.

### Oxygen-glucose deprivation procedure

To mimic an ischemic condition, oxygen-glucose deprivation (OGD) and reoxygenation were conducted. Briefly, a culturing medium was washed and replaced by no glucose Dulbecco’s Modified Eagle Medium (11966-025, Life Technologies). Cells were incubated at 37 °C in a humid chamber (Heidolph, incubator 1,000, Brinkmann Instruments,Westbury, NY) filled with an anaerobic gas (90% N_2_, 5% H_2_, and 5% CO_2_). OGD condition was aborted by changing an OGD medium to a culturing medium and further culturing cells in a normal incubator for reoxygenation.

### Cell viability assay

To measure the fraction of dead endothelial cells, cells were incubated in a culturing medium containing propidium iodide (PI) solution (P4864-10 ML, Sigma-Aldrich) at 1 mg.mL^−1^ (1:200) for 15 min at 37 °C. Then, cells were washed twice by PBS, fixed by 4% PFA for 15 min, and imaged in a mounting oil including a DAPI. To quantify the number of dead endothelial cells, we took fluorescent images, automatically counted the number of nucleus by using CellProfiler (Broad Institute, Boston, MA), and manually counted red-stained spots, which co-localizes with blue-stained nucleus.

## Additional Information

**How to cite this article**: Cho, H. *et al.* Three-Dimensional Blood-Brain Barrier Model for *in vitro* Studies of Neurovascular Pathology. *Sci. Rep.* **5**, 15222; doi: 10.1038/srep15222 (2015).

## Supplementary Material

Supplementary Information

Supplementary Movie 1

Supplementary Movie 2

Supplementary Movie 3

Supplementary Movie 4
