# Supplementary material for: Three-Dimensional Blood-Brain Barrier Model for in vitro Studies of Neurovascular Pathology
Source: Sci Rep. 2015 Oct 27;5:15222. doi: 10.1038/srep15222 (PMC4622078; doi:10.1038/srep15222)
Supplement: Supplementary Information [file srep15222-s1.doc]

**SUPPLEMENTARY INFORMATION**

Three-Dimensional Blood-Brain Barrier Model for
*in vitro* Studies of Neurovascular Pathology

Hansang Cho^1, 3^, Ji Hae Seo^2^, Keith H. K. Wong^1^, Yasukazu Terasaki^2^, Joseph Park^3^, Kiwan Bong^1^, Ken Arai^2^, Eng H. Lo^2^, Daniel Irimia^1*^

^1^BioMEMS Resource Center, Massachusetts General Hospital,
Harvard Medical School

^2^Neuroprotection Research Laboratory Center, Mass General Hospital,
Harvard Medical School

^3^Mechanical Engineering and Engineering Science
University of North Carolina at Charlotte

MATERIALS AND METHODS

**Gel staining.** To visualize gel formation, a FITC-conjugated collagen (C4361-10ML, Sigma-Aldrich) at 1 mg.mL^-1^ was used instead of a clear collagen type I. To avoid the decay of the signal by diffusion, the solution in the compartments was replaced by a collagen-repellant gel, Poly(ethylene glycol) (200) gel (P3015, Sigma Aldrich, St. Louis, MO) at 1 mg.mL^-1^.

**HUVEC preparation.** Endothelial cells (HUVEC: Human umbilical vein endothelial cells) were purchased from Lonza Inc. (Walkersville, MD, USA) and cultured in an endothelial medium, EBM-2 media containing 2% FBS, and other growth factors (0.04% Hydrocortisone, 0.4% hFGF-B, 0.1% VEGF, 0.1% R3-IGF-1, 0.1% Ascorbic acid, 0.1% hEGF, 0.1% GA-1000, 0.1% Heparin (Lonza Inc.)) and 1% Penicillin/streptomycin are used for cell culturing under 5% CO_2_ at 37 ºC. All HUVEC cells were used less than 7 passages.

**Immunostaining.** We rinsed the cells in medium twice with PBS, and then fixed them by incubating them in fresh 4% paraformaldehyde aqueous solution (157-4, Electron Microscopy Sciences, Washington, PA) for more than 15 minutes at room temperature. After fixing, cells were rinsed twice with PBS. To permeabilize the cells, we incubated them in 0.1% Triton X-100 in PBST (phosphate buffered saline with 0.1% tween®20) for 15 minutes at RT. To block, cells were incubated in 3% human serum albumin for overnight in PBST at 4 °C. To stain, cells were incubated with 1^st^ antibody for ZO-1 (339100, ZO-1, mouse monoclonal antibody, Invitrogen Corp., Camarillo, CA) at 5 *μ*g.mL^-1^, Ve-Cadherin (ALX-210-232, rabbit polyclonal antibody, Enzo Life Sciences, Inc., Farmingdale, NY) at 2 *μ*g.mL^-1^, and ROCK (ab2480, Anti-Myosin light chain (phospho S20) antibody, Abcam) at 2.5 *μ*g.mL^-1^ in PBST for overnight at 4 °C. Cells were incubated with 2^nd^ antibody for ZO-1 (715-586-151, Alexa Fluor 594-conjugated donkey antil-mouse IgG, Jackson Immuno Research Lab., West Grove, PA) at 1.5 *μ*g.mL^-1^, Ve-Cadherin (711-095-152, FITC-conjugated-donkey anti-rabbit IgG, Jackson Immuno Research Lab.) at 1.5 *μ*g.mL^-1^, and ROCK (711-586-152, Alexa Fluor® 594 AffiniPure F(ab')₂ Fragment Donkey Anti-Rabbit IgG, Jackson Immuno Research Lab.) at 5.0 *μ*g.mL^-1^ in PBST for three hours at 4 °C in a dark room. Solutions were rinsed twice with PBST after each step from permeabilization to 2^nd^ antibody incubation. To stain nucleus, rinsing PBST was replaced by mounting oil including a DAPI (17985-51, Fluoro-Gel II, Electron Microscopy Sciences) for 15 minutes at RT in a dark room before imaging. All incubation was performed in a humidity chamber.

**Rat Cytokine Array.** We employed a Rat Cytokine Array Kit (Proteome Profiler, ARY008 - R&D systems). First, membranes containing 29 different capture antibodies were incubated with blocking buffer for 1 hour. While the membranes were blocking, each 300 μl of medium was mixed with 15 μl of reconstituted Detection Antibody Cocktail and incubated at room temperature for 1 hour. We then added membranes with the sample/antibody mixtures, prepared in previous step, and incubated overnight at cold room on a rocking platform shaker. The next day, we removed each membrane and placed these into individual plastic containers with 20 mL of 1X Wash Buffer. We washed each membrane with 1X Wash Buffer for 10 minutes on a rocking platform shaker, repeating the procedure for a total of three washes. We diluted 1:2000 the Streptavidin-HRP and added 2.0 mL of diluted Streptavidin-HRP into each well. We incubated for 30 minutes at room temperature on a rocking platform shaker. We washed each array as described previously. We allowed excess Wash Buffer to drain from the membrane by blotting the lower edge onto paper towels. We then mixed 1 mL of the prepared Chemi Reagent Mix evenly onto each membrane and incubated for 1 minute. After carefully laying an absorbent lab wipe on top of the membranes, to blot off any remaining Chemi Reagent Mix, we exposed the membranes to Chemidoc reader for 1 minutes with 10 exposure times.

**ROS detection.** DCFDA Cellular ROS Detection Assay kit was purchased from Abcam (ab113851). We rinsed 3 times, first, with 50 μl of 1X buffer for each chamber. To stain cells, 1X buffer was removed and cells were stained by adding 50 μl of 20 μM DCFDA solution. And further we incubated cells for 45 minutes at 37^o^C and read the signal using fluorescence microscopy.

**FIGURE LEGENDS**

**Figure S1. Schematic representation of cell confinement by surface tension.** **a**. Introduced cells are plated on cell plating compartments by being confined with surface tension. **b**. Without the confinement, slightly uneven evaporation induces height difference of medium at reservoirs and consequently washing cells out of the plating compartments.

**Figure S2. Formation of a basal membrane in 3D**. Collagen type I of 2 mg.ml^-1^ fills PDL-coated (at 1 mg.ml^-1^) migration channels and coats a cell loading compartment. The formation of thin layer in the compartment as a basal membrane and solid gel in migration channels as ECM is validated by imaging FITC-labeled collagen in a confocal microscope. Scale bar, 50 *μ*m.

**Figure S3. Formation of endothelial monolayers in 3D and a large-scale.** Rat brain endothelial cells are plated and nucleus is stained with DAPI. **a**. Monolayer formation is validated by imaging cells on top/middle/bottom surfaces along straight (**1**) and curved compartments (**2**). Scale bars, 50 *μ*m. **b**. Monolayer formation is achieved in a large scale along the long cell compartment. Scale bar, 200 *μ*m.

**Figure S4. Inhibition of WBC transmigration by BBB model**. The inhibition of WBCs penetration is demonstrated by observing the reduced penetration of neutrophil (PMN, green) through the BBB (red) (**a**) compared to thorough penetration across HUVEC layer (**b**) and without the layer (**c**) along a gradient of a chemo-attractant, IL8. Scale bars, 50 µm.

**Figure S5. Reduced expression of ZO-1 proteins by TNF-α treatment.** The effect of TNF-α on BBB tightness is quantified by measuring the intensity profile of ZO-1 immunostaining. Localized ZO-1 around cellular boundary is discernable by being compared with average values indicated with dotted lines. The levels of ZO-1 decrease with the increase of the duration and the concentration of TNF-α.

**Figure S6. Slight effect of OGD treatment on the BBB viability**. **a**. Most of cells are alive without OGD treatment **b**. A few ECs turn to be dead after the OGD treatment for 40 minutes. Propidium iodide assay labels dead ECs in red. Scale bars, 50 *μ*m.

**Figure S7. Elevated level of ROS expression by ischemia condition**. Our BBB model treated by a combination of OGD for one hour and reoxygenation for three hours induces significant level of ROS expression (**a**) compared to control without any ischemia treatment (**b**). Scale bars, 50 *μ*m.

**Figure S8. Elevated level of ROCK expression by ischemia condition**. Our BBB model treated by a combination of OGD for one hour and reoxygenation for three hours induces significant level of p-MLC representing ROCK expression (**a**) compared to control without any ischemia treatment (**b**). Scale bars, 50 *μ*m.

**Figure S9. No toxicity of TNF-α treatment to the BBB**. TNF-α treatment has no effect on cellular viability and the measured viability remains at 99.5 % with the variation of TNF-α concentrations. Viability is quantified by propidium iodide assay.

**Figure S10. Significant effect of ischemia treatment on the BBB tightness**. An ischemia condition, combined treatments of OGD treatment and reoxygenation treatment significantly reduces the levels of ZO-1 expression and delocalizes the expression on the cellular boundaries from (**a**) to (**b**). OGD treatment for 1 hour and reoxygenation for 24 hours in (**b**). Scale bars, 20 *μ*m.

**Movie Legends**

**Movie M1. BBB-model inhibits transmigration of neutrophil toward IL-8 gradient.** Endothelial cells in red form tight membranes in the left compartment, which do not allow chemotactic migration of neutrophils in green even in the presence of chemo-attractant for neutrophils, IL-8 of 100 nM loaded in the right compartment. Scale bar, 200 *μ*m.

**Movie M2. Neutrophils migrate through migration channels along IL-8 gradient.** In the absence of the endothelial membranes, neutrophils migrate through collagen-filled channels aggressively and immediately along the primed gradient of chemo-attractant for neutrophils, IL-8 of 100 nM in the right compartment. Scale bar, 200 *μ*m.

**Movie M3. BBB-model of RBE4 endothelial cells inhibits transmigration of neutrophil toward IL-8 gradient (zoomed-in).** Several neutrophils might sense IL-8 and stayed on an endothelial membrane toward the source of IL-8 but could not cross the membrane during the two-hour experiment. Scale bar, 50 *μ*m.

**Movie M4. Neutrophils transmigrate across HUVEC endothelial layer along IL-8 gradient (zoomed-in).** Several neutrophils could find the weakly formulated region on an endothelial membrane and succeeded to migrate toward the source of IL-8. Scale bar, 50 *μ*m.


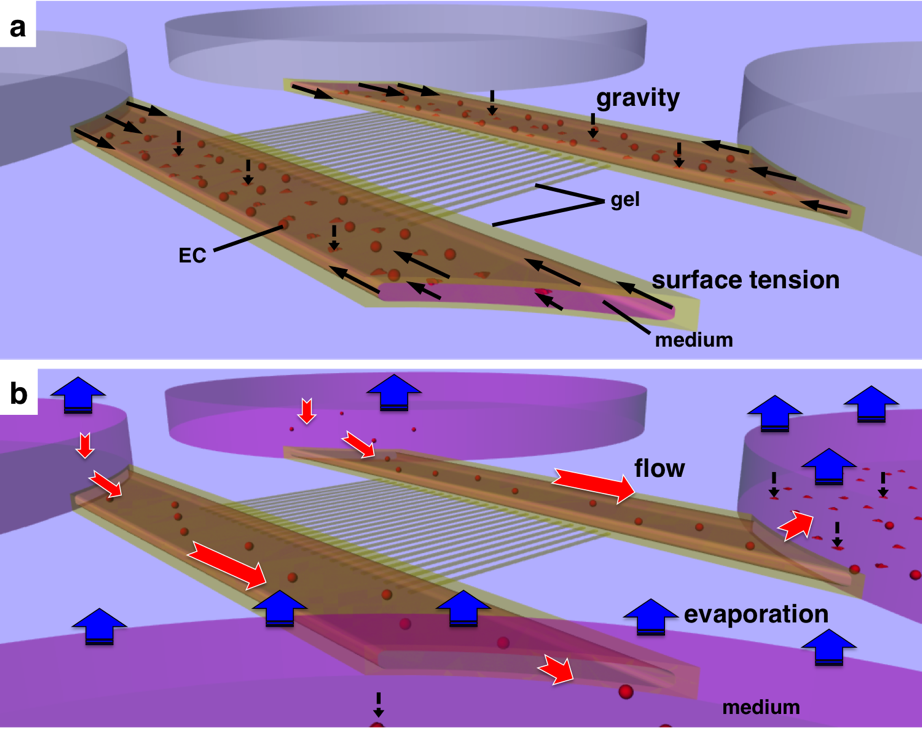


Figure S1.


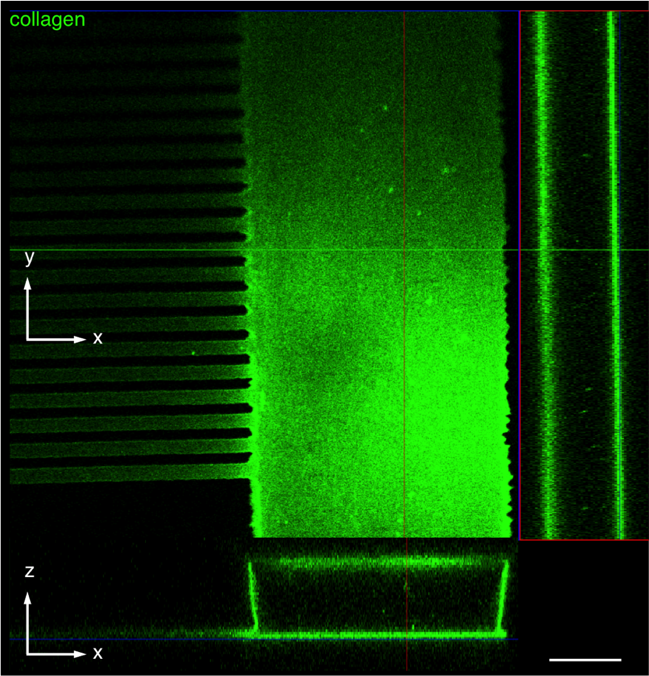


Figure S2.


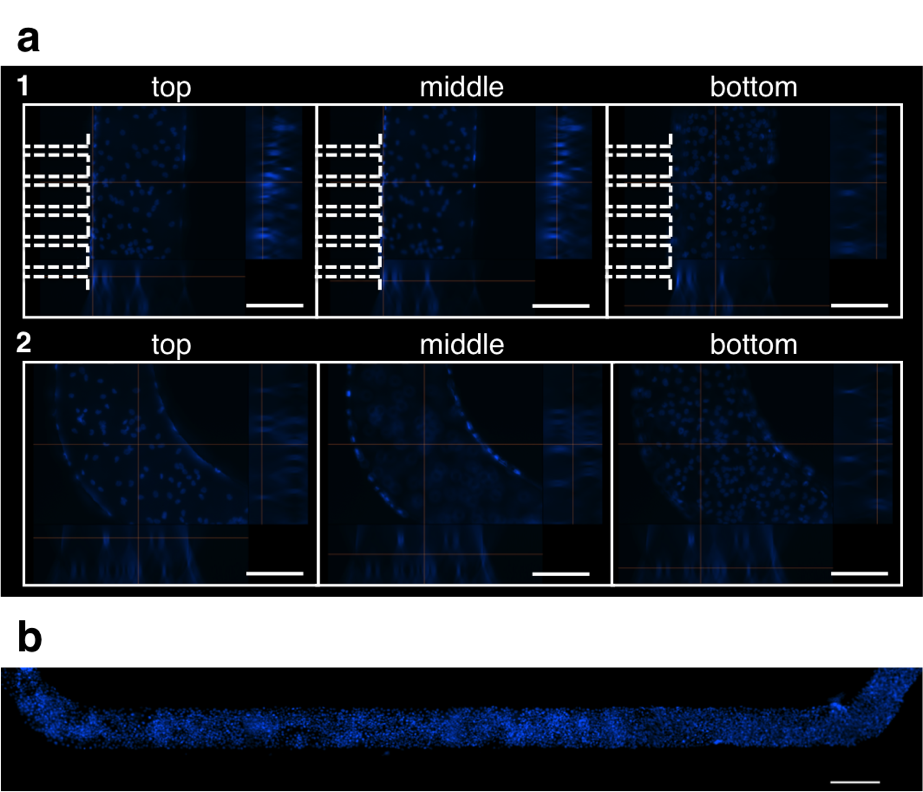


Figure S3.


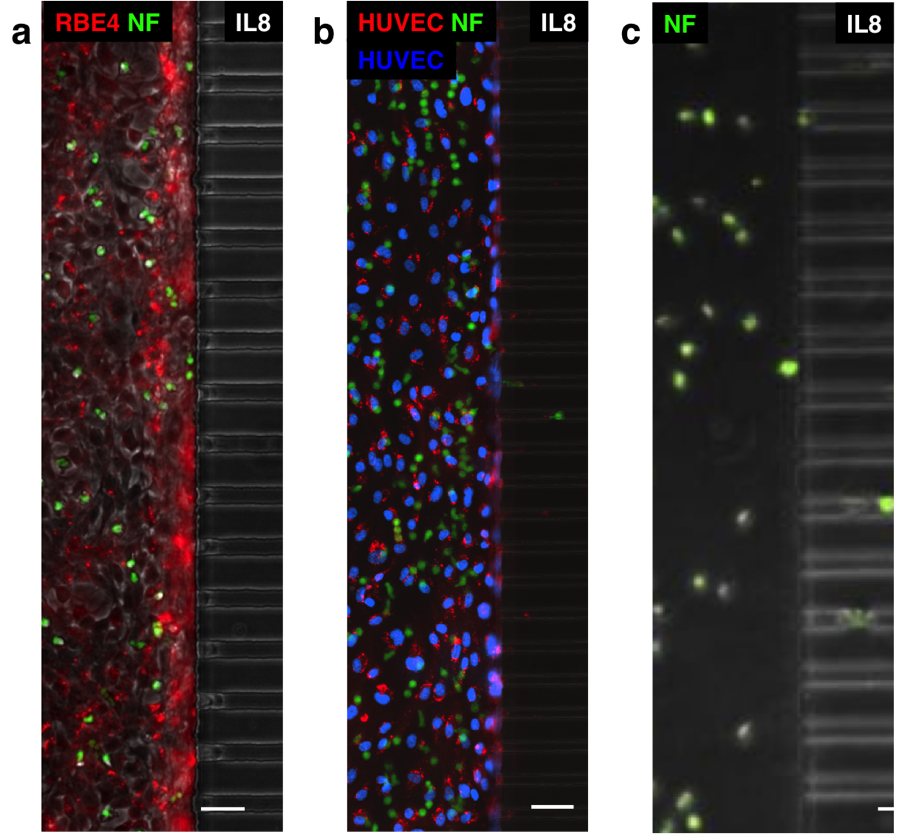


Figure S4.


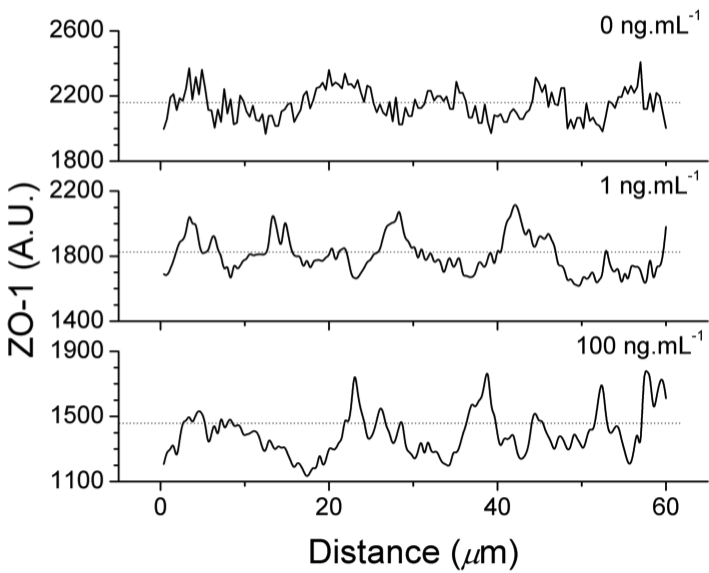


Figure S5.


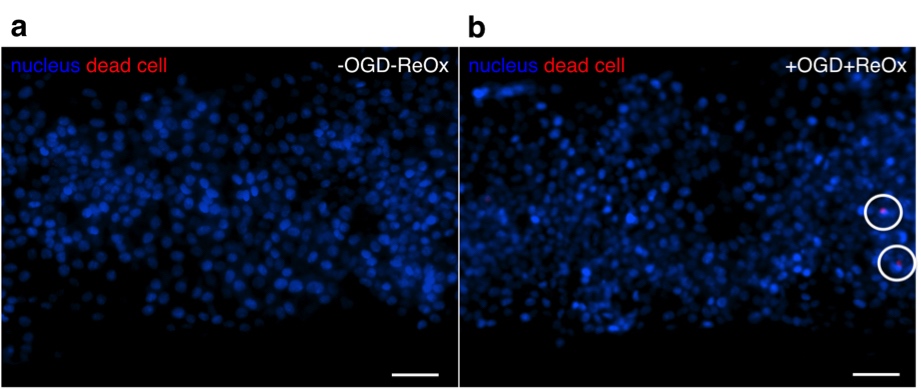


Figure S6.


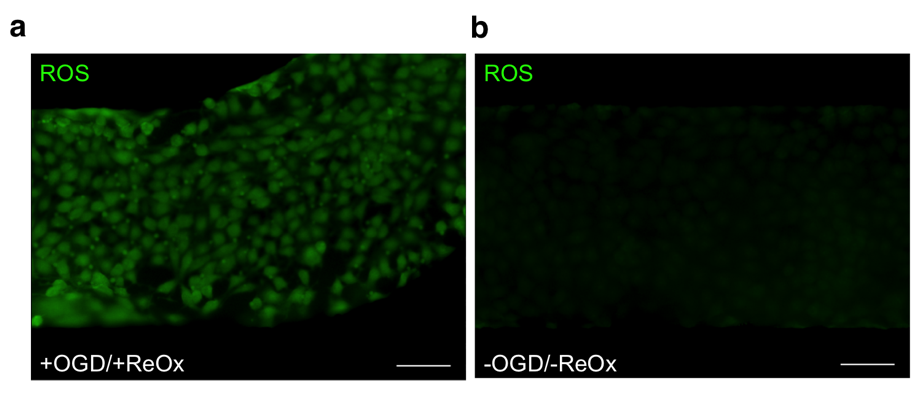


Figure S7.


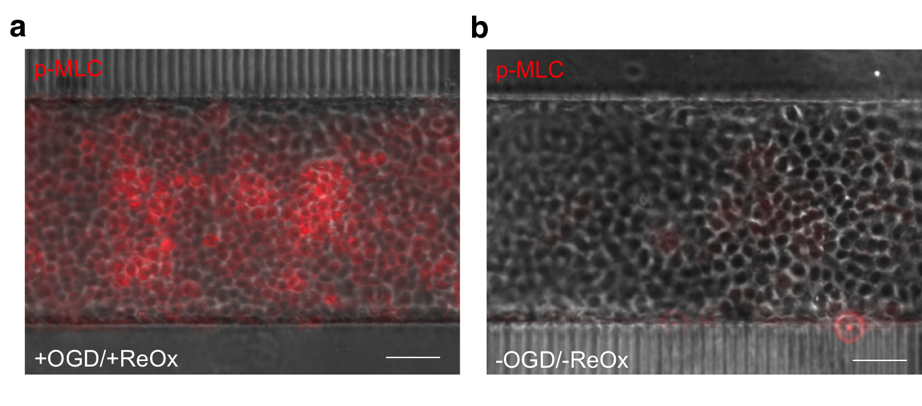


Figure S8.


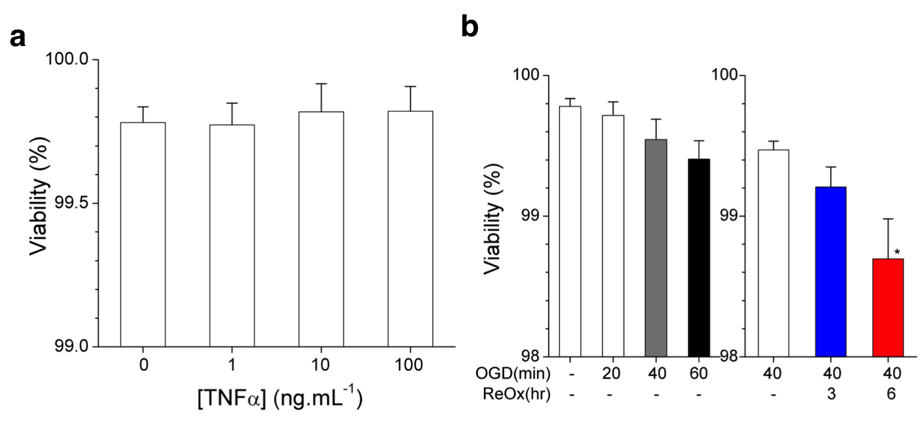


Figure S9.


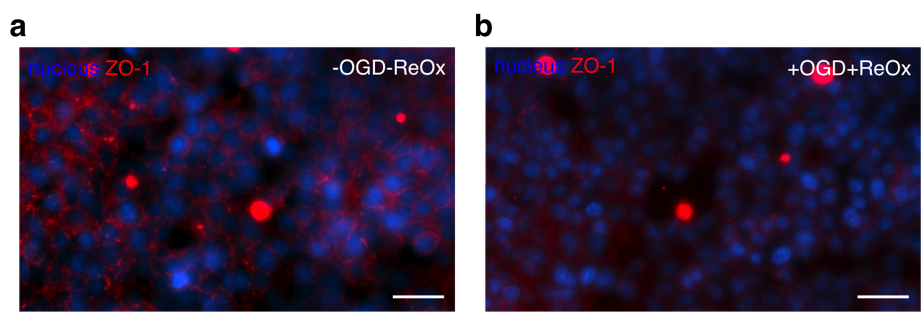


Figure S10.
